# Cognitive schemas and fertility motivations in the U.S. during the COVID-19 pandemic

**DOI:** 10.1553/populationyearbook2022.res1.7

**Published:** 2022-04-12

**Authors:** Wendy D. Manning, Karen Benjamin Guzzo, Monica A. Longmore, Peggy C. Giordano

**Affiliations:** 1 Department of Sociology, Center for Family and Demographic Research, Bowling Green State University, Bowling Green, OH, USA

**Keywords:** pandemic, fertility expectations, subjective appraisals

## Abstract

While current evidence indicates that the United States did not experience a baby boom during the pandemic, few empirical studies have considered the underlying rationale for the American baby bust. Relying on data collected during the pandemic (*n* = 574), we find that pandemic-related subjective assessments (e.g., self-reported stress, fear of COVID-19 and relationship struggles) and not economic indicators (e.g., employment status, income level) were related to levels of fertility motivations among individuals in relationships. Analysis of within-person changes in fertility motivations shows that shifts in the number of children, increases in mental health issues and increases in relationship uncertainty, rather than changes in economic circumstances, were associated with short-term assessments of the importance of avoiding a pregnancy. We argue for broadening conceptual frameworks of fertility motivations by moving beyond a focus on economic factors to include a cognitive schema that takes subjective concerns into account.

## 1 Introduction

Prior to the COVID-19 pandemic, the United States had been facing record declines in fertility levels (Hamilton et al., 2020); and current trends suggest that further declines are likely, as women have made downward adjustments in their fertility goals (Kahn et al., 2021; Lindberg et al., 2020; Luppi et al., 2020). Given the uncertain social and economic climate associated with this unprecedented pandemic, it appears that women and men may be adjusting their motivations, or schemas, regarding future childbearing and family life. Thus, the pandemic provides a critical opportunity to assess people’s fertility goals. We draw on the Toledo Adolescent Relationships Study (TARS), a population-based dataset with repeated measures of respondents’ fertility motivations prior to the pandemic (2018–2020) and during the pandemic (June–November 2020), to assess the fertility motivations of a sample of U.S. adults during the COVID-19 pandemic. The respondents are in their prime childbearing years, and we focus on those in relationships, for whom childbearing decisions are of more immediate relevance. We assess the respondents’ fertility motivations in the short term, and examine whether pandemic-related changes in their economic circumstances, relationships, health or stress levels have affected their fertility motivations. While building on prior demographic research on fertility, these key independent variables are not included in most demographic datasets, including in recent surveys. Furthermore, we examine changes in respondents’ fertility motivations both before and during the pandemic, and evaluate how changes in their number of children, health (physical and mental), economic circumstances and social ties have influenced their fertility motivations. These findings will help guide future research on the ways in which the pandemic has affected the lives of Americans, including their fertility behavior.

## 2 Background

It is well-documented that fertility levels in the United States are low, and concerns that the current low fertility levels may not rebound have been widely expressed in the media and in academic circles. Individual fertility preferences are responsive to societal shifts and pressures, including economic pressures (Hartnett and Gemmill, 2020). For example, fertility began falling around the time of the Great Recession (2007–2009), partly due to the disproportionate impact that this economic downturn had on individuals of childbearing ages (Cherlin et al., 2013; Percheski and Kimbro, 2017; Schneider, 2015; Su, 2019). Importantly, rates have continued to decline (Allred and Guzzo, 2018; Hamilton et al., 2020) despite an upturn in the economy after the recession. Generally, fertility falls during economic downturns. However, in the past, such fertility declines have tended to be brief, as postponed births are recouped after the economy has rebounded (Cherlin et al., 2013; Örsal and Goldstein, 2018; Sobotka et al., 2011). It appears, however, that the young adults who came of age during the Great Recession may not just be delaying, but may ultimately be reducing their fertility in response to the uneven economic recovery.

The U.S. total fertility rate (TFR) reached a decade low of 1.70 in 2019 (Hamilton et al., 2020), putting the United States on track to follow the path of many European countries. The factors that are associated with the extremely low fertility rates in European countries include the weak economic positions of young adults, low levels of economic and subjective well-being, and struggles to combine work and family obligations (Billari, 2018). Prior to the Great Recession, the United States had relatively high fertility – at or above 2.0 – compared to other industrialized nations. Now, however, the TFR in the United States is on par with that of nations with TFRs closer to 1.0, rather than with the pre-recession level of 2.0 (Billari, 2018). It is critical to understand how these downward changes occurred, especially as the COVID-19 pandemic continues to have social, economic and health impacts.

Examining fertility motivations can provide important insights into the processes that undergird aggregate fertility rates. At the most basic level, aggregate fertility trends are comprised of individual fertility decisions, and, on average, individuals’ fertility preferences tend to be strong predictors of aggregate fertility levels (Beaujouan and Berghammer, 2019; Morgan and Rackin, 2010). Unlike many other studies, we focus on short-term fertility motivations, as these are most likely to be directly affected by the changes that occurred during the pandemic. Men’s and women’s longer-term fertility goals may be relatively unaffected, given that both the modal category for the ideal family size and the average total intended parity in the U.S. has remained at two since before the Great Recession (Gemmill and Hartnett, 2020; Saad, 2018). Thus, people’s fertility behavior in the immediate future is more likely to be affected by the pandemic than their intentions to have children at some point over the longer term.^1^ Our focus on fertility motivations, and on how they are linked to various domains that reflect the context in which people make fertility decisions, will provide key insights into the factors that may be driving the fertility baby bust observed during the pandemic.

The societal implications of the pandemic are unlikely to represent a short-term *blip*. It is more likely that they will have an enduring impact, accelerating and exacerbating the declining fertility trends ushered in by the Great Recession. However, a unique feature that distinguishes the COVID-19 pandemic from prior economic downturns is the heightened sense of uncertainty brought on by the lack of a clear timeline regarding when, and if, American family life will return to *normal*; uncertainty regarding the potential long-term health effects of the pandemic beyond the risks of the disease itself; and uncertainty regarding employment and other changes in the economy (Calarco, 2021; Carlson, 2021; Landivar, 2021). This pervasive sense of uncertainty is not unwarranted. Rather, it is driven by individuals’ concerns about health care and medical treatments, skyrocketing unemployment levels, shifting workplace demands and increases in parenting obligations in the face of child care and school closures, among other factors. Cumulatively, these concerns are challenges for couples in intimate relationships, and constrain individuals’ social lives in new ways. Americans of childbearing age have not previously faced so many forms of sustained uncertainty, and at such high levels, in their lifetimes. Thus, it seems quite likely that the current climate characterized by pervasive uncertainty will further dampen fertility motivations. Indeed, in Europe, about 70% of respondents who planned to have a child during 2020 reported either postponing or abandoning their plans during the pandemic (Luppi et al., 2020). However, no consistent pattern tying fertility decisions to perceived income declines or the spread of COVID-19 has been observed.

Claims that there have been pandemic-related changes in people’s life circumstances are often based on limited, but compelling survey evidence. People’s economic concerns are evident based on their responses to the recession and skyrocketing unemployment rates (BLS, 2021), as well as their expressions of pessimism about their financial future (Parker et al., 2021). It certainly appears that the pandemic has caused people to pay increased attention to their health, both currently, and over the long term (Dayton et al., 2021). There is evidence indicating that the pandemic has led to changes in Americans’ social and psychological well-being, including increases depressive symptoms and anxiety over time (Ettman et al., 2021; Manning et al., 2021). Similar findings have been reported in cross-sectional population-based surveys (Jia et al., 2021). The empirical literature on stress related to COVID-19 has shown that nearly 40% of individuals have reported experiencing some distress during the pandemic (Taylor et al., 2020). On the relationship front, it appears that there have been challenges to relationship functioning during the pandemic. There is, for example, evidence suggesting that there have been short-term changes in the prevalence of relationship conflicts, but limited shifts in the numbers of physical fights over a one-month period (Lee et al., 2021). While such findings are not conclusive, it appears that there have been substantial shifts in key dimensions of well-being during the pandemic, which may have affected fertility decision-making.

We argue that traditional theoretical approaches focusing on planned behaviors may not be relevant during periods characterized by substantial uncertainty. With regard to the formation of childbearing decisions, greater conceptual attention to the link between plans and outcomes is needed. To better understand how the confluence of pandemic-related changes and stressors have affected fertility, we draw on insights from the Theory of Conjunctural Action, or TCA (Johnson-Hanks et al., 2011). Central to the TCA is the assumption that individuals’ “schemas” – i.e., their ideas, values, beliefs, scripts and patterns of thinking – inform and guide their behavioral intentions and actions. The TCA provides a framework for conceptualizing the new reality in the United States, which is characterized by the lack of a clearly outlined and predictable future, by drawing attention to the schemas that people use to make sense of a situation, and to inform their decision-making, including their fertility decisions. During the pandemic, the heightened sense of uncertainty surrounding health, economic, relational and childrearing decision-making has meant that individuals can no longer rely on their past experiences (typically thought of as the best predictor of future behavior) (Ferrante et al., 2013; Ouellette and Wood, 1998) or pre-existing attitudes to guide their decisions. In brief, what was previously “true” or “right” may no longer be applicable. Women and men facing such high levels of uncertainty are likely to hold off on making any new commitments, including having a child, until they have a better grasp on their lives, and on the situations that they will face in the future.

The TCA has been applied to empirical research on fertility in Africa and Europe that focuses on the context of uncertainty (e.g., Hayford and Agadjanian, 2011; Trinitapoli and Yeatman, 2011). Moreover, consistent with this theoretical approach, scholars focused on Europe have also have called for theoretical developments (e.g., Narratives of the Future) that address how uncertain economic contexts influence fertility decisions (Vignoli et al., 2020a). Vignoli and colleagues found that employment uncertainty influenced fertility intentions through indicators of well-being (Vignoli et al., 2020b); and that perceived uncertainty at the macro level due to the debt crisis influenced fertility (Comolli and Vignoli, 2021). We extend this focus on uncertainty to the current situation in the United States by including indicators that reflect economic, health and relational uncertainties; and by assessing whether such uncertainties affect fertility schemas. For example, in the United States, a normative schema surrounding the decision to have a child is that childrearing requires major economic, emotional and social investments, which directly affect children’s development, and, ultimately, their life success (see e.g., Blair-Loy, 2009; Bock, 2000; Calarco, 2018; Hays, 1998; Lareau, 2011; Myers, 2017). As a consequence of the uncertainties caused by the pandemic, the strength or certitude of this schema has likely been amplified. Furthermore, during certain phases of the pandemic, the burdens of parenting were shouldered almost entirely by parents, as child care centers and schools remained closed, or opened only intermittently (Landivar, 2021). Moreover, due to social distancing mandates, parents could no longer rely on their social networks for child care, emotional support and social activities. Earlier research drawing on the TCA has found that fertility preferences are responsive to “contingencies, inputs and shifts that occur in micro and macro levels” (Trinitapoli and Yeatman, 2018, p. 87), and this conclusion has been supported by recent empirical evidence (e.g., Hartnett and Gemmill, 2020). Building on these prior studies, we expect to find that indicators of uncertainty are associated with decreased fertility plans. However, unlike prior studies, our data permit us to focus on multiple domains of uncertainty, including health, relationship and economic concerns associated with the COVID-19 pandemic.

In addition, we move beyond prior work by focusing on pregnancy avoidance, rather than on wanting/planning to get pregnant. This approach reflects the hypothesized theoretical links: i.e., that the pandemic affected people in ways that made childbearing less desirable over the short term. While it could be argued that changes during the pandemic might have made it less necessary to avoid a pregnancy (for instance, working from home may have alleviated parental leave or child care concerns, thus reducing work-family conflict), it is harder to make the case that the pandemic increased the sense of urgency about having a child. Again, given that most Americans want small families, there is generally little or no urgency to have a child at a particular point in time to achieve their fertility goals; indeed, most people of reproductive age spend the majority of their fertile years actively avoiding a pregnancy. Pregnancy avoidance measures have been widely used in studies of fertility and reproductive behavior to capture pregnancy intentions and desires (e.g., Barber et al., 2019; Hayford and Guzzo, 2013; Higgins et al., 2012).

The overarching goal of the current study is to provide theoretically-informed insights into fertility more broadly. Although there is early evidence of a decline in fertility during the pandemic (e.g., Cohen, 2021; Sobotka et al., 2021), full vital statistics data on fertility in the United States during this period will not be available until 2022. Early data for the first quarter of 2021 are available for two states, and suggest that there was a decline in “pandemic babies” (Cohen, 2021). However, the evidence regarding births in the rest of the nation, and into the summer and fall, is inconclusive. In addition to vital statistics data, another major source of data on fertility goals and behaviors in the United States is the National Survey of Family Growth, which also will not release its data covering this period until roughly 2022. While some organizations have fielded surveys to investigate fertility preferences and behaviors, there is a pressing need for more timely research. Still, the limited data that are available have demonstrated that the pandemic has indeed led to shifts in fertility decision-making. For example, the Guttmacher Survey of Reproductive Health Experiences found that two-fifths of women of reproductive age have changed their fertility plans in response to the pandemic (Lindberg et al., 2020). Extending existing descriptive profiles, this study uses population-based data that cover periods before and during the pandemic to assess how people’s fertility motivations developed and changed during these critical periods.

## 3 Current study

Despite speculation in the media about a COVID-19 baby boom, it is fairly clear now that there was no such baby boom, and that there was instead a baby bust. We add to this straightforward conclusion by providing empirical evidence on the mechanisms underlying this fertility decline, and thus seek to shed light on the question of why the United States did not experience a baby boom. In our first research question, we hypothesize that economic, relational and health uncertainties dampened fertility motivations during the pandemic. Specifically, we focus on a measure of pregnancy avoidance, because it reflects whether childbearing became less desirable over the short term, and because it is consistent with the observation that most of adulthood is spent avoiding having children (Barber et al., 2019; Hayford and Guzzo, 2013; Higgins et al., 2012). Unlike some demographic research that has relied on unmeasured indicators that are implied based on behaviors or contextual measures, our analyses include direct measures of uncertainty. The data include pandemic-related subjective assessments (e.g., self-reported stress, fear of COVID-19, relationship struggles) as well as behavioral indicators (e.g., employment status, income level). Importantly, because Americans’ responses to the pandemic may have been colored by political ideology, we consider whether respondents expressed approval of the government’s handling of the pandemic, and whether they agreed with the statement that the media are overreacting to the pandemic. The second research question utilizes the longitudinal TARS data to assess changes in the importance placed on avoiding a pregnancy. We hypothesize that the number of children, health status, economic circumstances, parental attachment and relationship certainty/uncertainty were associated with the importance placed on avoiding a pregnancy. We expect to find that parents were especially likely to report that they consider avoiding having another child to be important.

## 4 Data

The TARS is a study of the lives of a diverse sample of adolescents (*n* = 1,316) who were interviewed seven times as they transitioned to adulthood (2001, 2002, 2004, 2006, 2011, 2019, 2020). The sixth wave of data collection was conducted April 2018–March 2020, and included 990 respondents who were aged 29–36 (mean age of 32). Although the sample, which was devised by NORC (National Opinion Research Center), was initially based on school rosters, school attendance was not a requirement for inclusion. Thus, the sample included young adult women and men who represented a broad range of socioeconomic circumstances. The population-based sample was regional; nevertheless, the respondents were demographically similar to 30–34-year-olds at the national level when compared to the American Community Survey data (e.g., in the TARS sample, 38% of respondents were racial/ethnic minorities, compared to 35% of the U.S. population; and 36% of respondents were college graduates, compared to 40% of the U.S. population). In response to the pandemic, new data, wave 7, were collected with a brief (25-minute) online survey that afforded a unique opportunity to assess behaviors and attitudes during the pandemic. High response rates have been maintained; for example, between waves 6 and 7, we retained 82% of the sample. Overall, the characteristics of the wave 7 sample differed somewhat from those of the wave 1 sample due to attrition, with attrition being greater among men and racial and ethnic minorities in the wave 7 sample. The interviews were conducted between June and November 2020. During this time period, which was prior to the release of vaccines, Americans were experiencing a high degree of uncertainty about the course of the pandemic. While the respondents in the sample were spread across 41 states and U.S. overseas territories, the majority were living in Ohio. During this time period, Ohio was experiencing elevated COVID-19 infection rates and hospitalizations, but the state had not yet reached peak COVID-19 mortality levels.

To ensure consistency across our analyses, the analytic sample included women and men who answered both surveys (*n* = 815) and reported valid data on fertility expectations (*n* = 756). The results are focused on a sample of 574 respondents who were in dating, cohabiting or married relationships at wave 7. This restriction excluded respondents who were most motivated to avoid having children because they were not in a relationship, and may not have been exposed to the risk of having a child (as they may have had no sexual relationships). Supplemental analyses were conducted that included respondents who were single at wave 7 (*n* = 756), and separate analyses were conducted among respondents who reported being with the same partner at both waves 6 and 7 (*n* = 494). Sensitivity checks indicated that restricting the sample to respondents with valid wave 6 data on their fertility motivations did not influence the levels of fertility motivations observed in wave 7.

### Dependent Variable.

The dependent variable was based on data on the fertility motivations reported at wave 7, with some preliminary analyses of data on the levels reported at wave 6. In wave 6 and in the wave 7 COVID-19 module, respondents’ *fertility motivations* were measured using the following question: “How important it is to avoid becoming pregnant right now?” The responses were provided on a five-point scale ranging from “not at all important” to “very important.” Respondents were asked about their immediate motivations because the aim of the item was to assess the impact of the pandemic on their current circumstances, and not at an unspecified time in the future.

### Pandemic-Related Independent Variables.

The key independent variables for the analyses of fertility motivations during the pandemic were based on pandemic-related indicators. The *fear of COVID-19* variable was based on two items that assessed the frequency of the following worries: (1) “Worried that you might contract the virus” and (2) “Worried that one or more members of your family might contract COVID-19” (alpha = .86). Responses were provided on a five-point scale ranging from “never” to “often.”

*Conservative political beliefs* were measured as the level of agreement with the following two items: (1) “Politicians, the news and other social media have exaggerated the risk” and (2) “The government should not tell me what to do” (alpha = .74). The possible responses ranged from (1) “strongly disagree” to (5) “strongly agree.”

Among the potential sources of support during the pandemic were the respondents’ own parents. The *parental arguments* variable was based on a single question: “How often do you and your parents have arguments about issues related to social distancing or COVID-19?” The response options ranged from “never” to “very often.” The aim of this question was to assess respondents’ levels of agreement or disagreement with significant others regarding compliance with health mandates that may be perceived as challenging.

The variable on *relationship uncertainty* – i.e., uncertainty about the relationship with the current partner – was based on a single item. Respondents were asked about the extent of their agreement with the following statement: “Our relationship feels more uncertain than ever.” The possible responses ranged on a five-point scale from “strongly disagree” to “strongly agree.”

The *work from home* variable was based on responses to the question of whether the respondent or his/her partner had started working from home during the pandemic. The response categories were “yes” and “no.”

The variable on *loss of income* due to the pandemic was based in part on affirmative responses to the item: “Since the COVID-19 pandemic occurred how much has your income from all sources been affected?” The response options included “much less income” or “somewhat less income.” The variable was also based or affirmative responses to the question of whether the respondent or his/her partner “experienced a cut in pay as the result of the COVID-19 pandemic.” An additional variable measuring *employment change* was based on responses to questions about whether the respondent or his/her partner was employed at the time of the interview, and whether s/he had been employed prior to the pandemic. Affirmative responses indicated that the respondent had been “laid off” or “furloughed.” We also logged the respondents’ *household income* at wave 6. (These latter two measures were not included in the final models, as they were not associated with fertility motivations). *Stress* was measured based on a single item: “*Since COVID-19 how stressed have you been due to your future?*” Responses were provided on a five-point scale ranging from “not at all stressed” to “very stressed.”

### Independent Variables Included in Models of Changes in Fertility Motivations.

The independent variables in the analyses of changes in fertility motivations were aligned with the pandemic-related factors (presented above), including changes in fertility, economic, health and social ties that occurred before and during the pandemic. These indicators were measured in the same way at both interview waves. The variable on the *change in number of children* was based on questions about the number of biological children, and ranged from zero to four, with 73% of respondents reporting no change.

The variable on changes in *economic hardship* was based on six questions with “yes” and “no” responses, including items that asked respondents whether they “didn’t pay the full amount of the mortgage or rent because there wasn’t enough money” or “couldn’t see a doctor or go to hospital because there wasn’t enough money.” The possible responses ranged from “strongly disagree” to “strongly agree.” The changes in the hardship indicator ranged from −5 to five, with 61.7% reporting no change, 25.3% reporting fewer hardships and 13.1% reporting more hardships. *Economic stress* was measured based on two items posed at each interview wave: “How stressed have you been about money/finances?” and “How stressed have you been about work/employment?” Responses were given on a five-point scale. The indicator ranged from −4 to three, with 22.5% of respondents reporting no change in their economic stress, 44.4% reporting less stress and 33.1% reporting increased stress.

The self-reported *physical health* indicator was based on a question that asked respondents about potential changes in their health. Responses were provided a five-point scale ranging from “poor” to “excellent.” The indicator ranged from −2 to three, with 54.5% of respondents reporting no change in health, 21.9% reporting declining health and 23.6% reporting improved health.

The *mental health* of respondents was measured based on their self-reported depressive symptoms using an eight-item version of the CES-D scale (Radloff, 1977). The respondents were asked how often each of the following statements had been true over the past week: (1) “You felt you just couldn’t get going;” (2) “You felt that you could not shake off the blues;” (3) “You had trouble keeping your mind on what you were doing;” (4) “You felt lonely;” (5) “You felt sad;” (6) “You had trouble getting to sleep or staying asleep;” (7) “You felt that everything was an effort;” and (8) “You felt depressed.” Higher scores indicated higher levels of depressive symptoms, and ranged from one (“never”) to eight (“every day”). The summed scale ranged from eight to 64. The indicator on changes in depression ranged from −56 to 47, with 11.7% of respondents reporting that there was no change in their depressive symptoms, 28.6% indicating that their depressive symptoms had decreased and 59.7% reporting that their depressive symptoms had increased.

*Closeness to parents* was assessed based on the level of agreement (“strongly disagree” to “strongly agree”) with a single item: “I feel close to my parents.” While 65.3% of respondents indicated that their closeness to their parents had not changed, 16.7% reported experiencing less closeness and 18.0% reported experiencing more closeness.

*Relationship uncertainty* was measured with two items: “I feel uncertain about our prospects to make this relationship work for a lifetime” and “I would leave my partner if it was not so difficult to do so.” The potential responses ranged from “strongly disagree” to “strongly agree.” The alpha on this indicator was 0.82 before the pandemic and was 0.79 during the pandemic. The indicator on changes in relationship uncertainty ranged from −8 to seven, with 46.7% of respondents reporting no change, 36.8% reporting less uncertainty and 17.5% reporting greater uncertainty.

### Sociodemographic Characteristics.

Six sociodemographic indicators were included in the analysis of fertility motivations during the pandemic. *Parenthood* was a dichotomous measure indicating whether the respondent had biological children at wave 7. *Gender* was coded as 1 = female and 0 = male. *Race*/*ethnicity* was recoded into four categories: non-Hispanic white (reference category), non-Hispanic Black, Hispanic and “other.” *Education* was measured at wave 6, and was based on the respondents’ highest level of education: high school (or less) (reference category), some college and college or more. Union status at wave 7 was measured using three categories: dating (reference), cohabiting and married. *Age* was measured in years using a continuous variable based on the respondents’ reported age at wave 7. To account for the rapid changes in the pandemic over time, a series of dummy variables indicating the *month of interview* (June-October/November) were included, but were not shown in the models.

## 5 Analytic strategy

For the first research question, we analyzed how pandemic-related indicators were associated with respondents’ fertility motivations during the pandemic. We used OLS regression modeling to estimate the association between pandemic-related measures and sociodemographic characteristics, and to assess how these indicators influenced the desire to avoid pregnancy.

The second research question analyzed changes in respondents’ fertility desires across interview waves; i.e., before and after the start of the pandemic. Using fixed-effects regression models (Allison, 2009), we examined how changes in fertility, economic factors, health, social ties (parents and partner) and levels of depression were associated with changes in motivations to avoid pregnancy before and during the pandemic. We pooled pre-pandemic and pandemic data from the TARS, and estimated fixed-effects models by examining how changes in economic, relationship and health stressors; uncertainty about the future; and fertility were associated with changes in fertility expectations. One advantage of fixed-effects modeling is that it uses each individual as his/her own control, and thus statistically removes unobserved, time-invariant variables that may confound the association between key predictors and fertility motivations (i.e., reducing endogeneity).

## 6 Results

### 6.1 Fertility motivations during the pandemic

Table 1 presents the distribution of the analytic sample. The mean response for the question on the importance placed on avoiding a pregnancy was 3.14, or “somewhat important.” During the pandemic, about two-fifths of respondents reported that they considered avoiding a pregnancy to be very important; while 30.5% of respondents reported that they viewed avoiding a pregnancy as not important at all.

The multivariate ordinary least squares regression results estimating the importance placed on avoiding a pregnancy during the pandemic are presented in Table 2. (Given the skewed distribution of the dependent variable, a logistic regression estimating the importance placed on avoiding a pregnancy was also tested, and similar results were obtained.) The sociodemographic characteristics of respondents were not strongly associated with how important they considered avoiding a pregnancy to be. On average, parents reported a stronger desire to avoid a pregnancy than respondents who were not yet parents. Men and women were roughly equally likely to want to avoid a pregnancy. Latinx or Hispanic respondents were less likely to want to avoid a pregnancy. The remaining measures of education, union status and age were not associated with the desire to avoid a pregnancy.

The next set of indicators addressed pandemic-specific factors. Respondents who were worried about themselves or their family members getting COVID-19 reported having a stronger desire to avoid a pregnancy. The pandemic-related political views of respondents were not associated with their fertility motivations. With regard to social ties, whether respondents were arguing with their parents about social distancing was not significantly associated with their fertility motivations. In contrast, the respondents’ relationship context was associated with the importance they placed on avoiding a pregnancy; i.e., respondents who were more uncertain about their relationship since the start of the pandemic had a greater desire to avoid a pregnancy. Contrary to expectations, respondents’ economic indicators were not associated with their pregnancy motivations. Working from home was associated with a stronger desire to avoid a pregnancy at the bivariate level (not shown), but not in the multivariate model. Having experienced a loss of income was not associated with the importance placed on avoiding a pregnancy. In an effort to determine whether our economic indicators were or were not capturing the respondents’ economic stresses and strains, we conducted supplemental analyses that included changes in employment (not working, laid off or furloughed) as well as household income; and the results showed that neither were associated with the importance placed on avoiding a pregnancy (results not shown). Finally, respondents who reported feeling more stressed about their future tended to place greater importance on avoiding a pregnancy. In sum, the results showed that having relationship-based problems and feeling stressed about the future were more strongly related to fertility motivations than to economic factors.

It is, of course, possible that economic factors drove the respondents’ feelings of stress about their relationship or the future. To delve further into the role of economic factors, we conducted supplemental analyses to determine how economic indicators influenced the respondents’ levels of stress, fear of COVID-19 and relationship uncertainty (results not shown). The results suggest there may have been an indirect pathway through which economic indicators influenced the respondents’ fertility motivations during the pandemic.

Finally, as the analytic sample was limited to individuals who were in a relationship at wave 7, we conducted supplemental analyses that included respondents who were single (not dating, cohabiting or married) at wave 7. Respondents who were in a relationship reported placing less importance on avoiding a pregnancy than single respondents did. The multivariable results on single respondents’ views on pandemic-related measures (relationship uncertainty was excluded from the model) were similar to those of the partnered respondents, with one exception. In this model, concerns about COVID-19 were not associated with fertility motivations, which could be partly because single respondents were less worried than partnered respondents about COVID-19.

### 6.2 Changing fertility motivations

**Figure 1:**
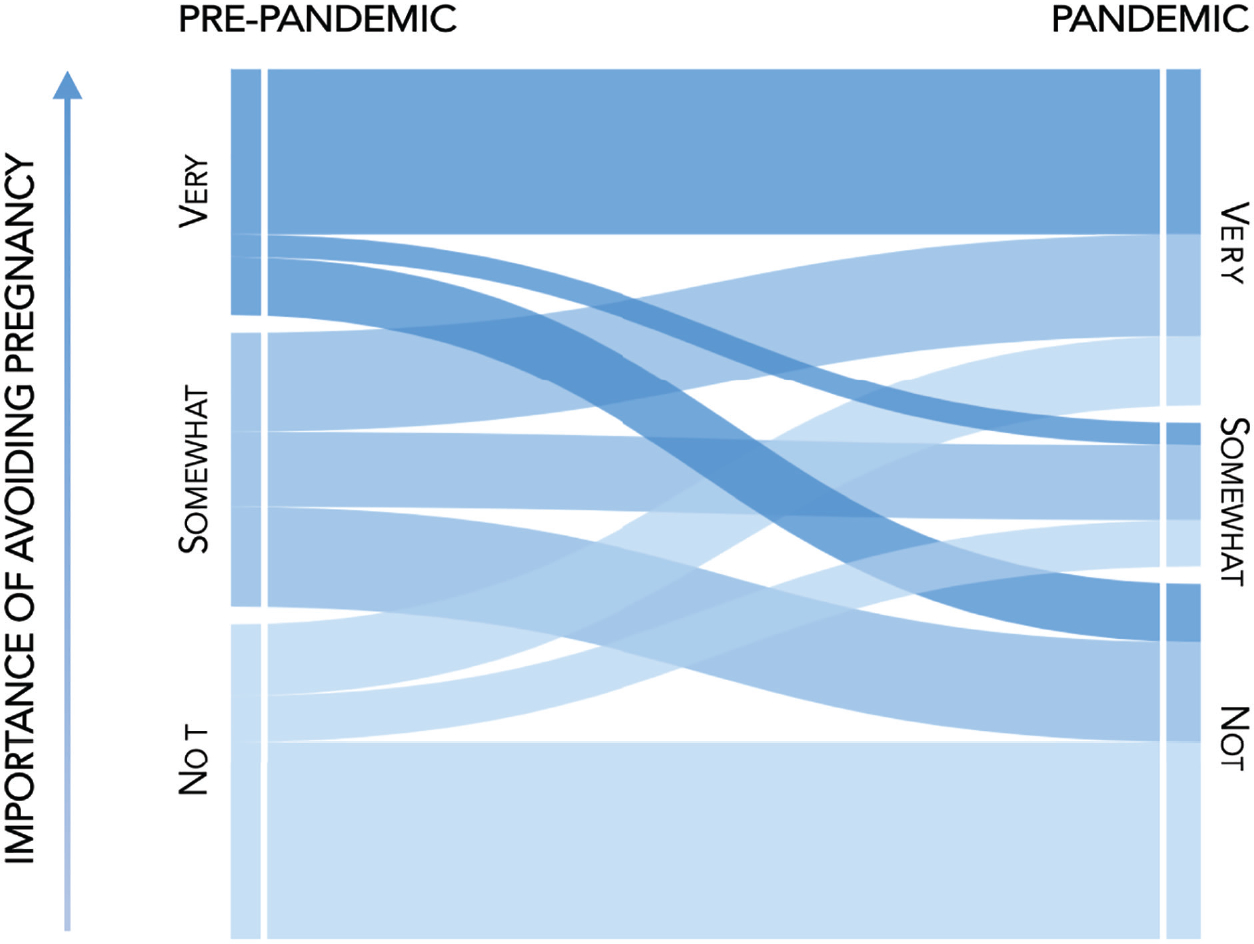
Continuities and changes in the importance placed on avoiding a pregnancy before and during the pandemic

The next research question assessed changes in the importance placed on avoiding a pregnancy. Figure 1 presents the changes in responses from the period before to the period during the pandemic. To simplify the figure, the measures of importance were categorized into three groups: not too important or not important; somewhat or fairly important; and very important. It is clear that there were both continuities and changes in the importance placed on avoiding a pregnancy. While there were flows in both directions, there was a significant (*p* = .000) increase in the percentage of respondents who reported that they considered avoiding a pregnancy to be very important, from 29% before the pandemic to 40% during the pandemic. Notably, about one-quarter of respondents ported that they viewed avoiding a pregnancy as not important at both time points.

Table 3 presents the distribution of the indicators used in the fixed-effects models of changes in fertility motivations. On average, respondents’ fertility motivations underwent only modest changes across the interview waves. As expected, respondents had, on average, more children across the interview waves. Moreover, between the interview waves, economic hardship declined, but economic stress increased, on average. The changes in physical health were minimal, but mental health issues increased across the interview waves. The mean level of parental closeness did not change between the interview waves, and the mean level of relationship uncertainty declined.

The coefficients in the fixed-effects models estimating changes in fertility motivations were quite similar to those estimating the levels at wave 7 (Table 4). These models required indicators that were identically measured at both interview waves, and were linked to the pandemic-based indicators. The model indicated that an increase in the number of children was associated with placing greater importance on avoiding a pregnancy. Shifts in levels of economic well-being, economic hardship and stress about work or money were not associated with changes in fertility motivations. Moreover, changes in self-rated health were not linked to changes in fertility motivations. With regard to social ties, changes in levels of closeness to parents were not associated with shifts in fertility motivations. Respondents who indicated that they were more uncertain about their relationship also reported an increased desire to avoid a pregnancy. Finally, an increase in self-reported depressive symptoms was associated with a greater desire to avoid a pregnancy. Supplemental analyses indicated that when the analytic sample was limited to individuals who were in the same relationship at both waves (*n* = 494), the results were similar (results not shown).

## 7 Discussion

The pandemic has fundamentally changed how individuals live their lives. Although it remains to be seen which of these changes become permanent as society slowly, and fitfully, recovers from the pandemic, there is little doubt that these changes have introduced new stressors and sources of uncertainty to wide swaths of the population, and have had ripple effects that go well beyond those related to health. In this paper, we considered how the pandemic, and the shifts in personal, relational and economic well-being that accompanied it, influenced the fertility motivations of individuals in their childbearing years using longitudinal data that are uniquely suitable for comparing individuals’ fertility plans – as well as their status and overall well-being– before and after the start of the COVID-19 pandemic.

Our approach was grounded in the Theory of Conjunctural Action, which argues that individuals draw on established mental schemas to make sense of, and to respond to, events and situations. Among Americans, the normative cognitive schema regarding childbearing centers around the notion of what children need from parents to succeed. In this schema, parents and would-be parents consider whether they have the resources – e.g., economic and relational stability, social support from personal networks, stable housing and employment and safe and reliable child care – to provide for children, and to maximize their chances of success (e.g., Blair-Loy, 2009; Bock, 2000; Calarco, 2018; Hays, 1998; Lareau, 2011; Myers, 2017). Furthermore, there is an ongoing dialogue not just about the direct costs of childrearing and its impact on employment (especially for mothers); but also, via social media, about the opportunity costs of childrearing in terms of leisure time, and the challenges of parenting (Orton-Johnson, 2017). Given that levels of uncertainty have increased across multiple domains, even as levels of concern about how the challenges associated with raising a child could affect the well-being of both the parents and the child have grown, finding a schema for making sense of the pandemic is likely to be a problem for many men and women of childbearing age. As such, we anticipate that pandemic-related fears and uncertainty will lead many people to avoid childbearing in the near future.

Although there is emerging evidence that fertility rates have indeed declined during the first quarter of 2021, the specific mechanisms that drove these lower birth rates are unclear. In particular, given the cascading sets of changes across domains, identifying which factors – for instance, economic concerns or stress within intimate partnerships, or health-related fears – is challenging. However, to design interventions aimed at stemming, if not reversing, ongoing fertility declines, it is necessary to identify these factors. In this paper, we explored the desire to avoid having a child among a longitudinal sample of men and women. The results showed that in summer or fall of 2020, about four in 10 adults aged 31–38 (mean age of 34) in a relationship reported that they considered avoiding a pregnancy to be important, up from about three in 10 prior to the pandemic.

We had expected to observe that experiencing uncertainty and stress increased the likelihood of wanting to avoid a pregnancy, and our results largely supported this expectation. Specifically, we found that partnered men and women who reported being more afraid of COVID-19, more stressed about the future and more uncertain about their relationship also reported a stronger desire to avoid a pregnancy. There was, however, one interesting exception to this general pattern. Unexpectedly, and inconsistent with the cognitive schema of needing to feel financially settled before having children, we did not find that economic factors directly influenced the desire to avoid a pregnancy. This finding held true even when we tested a fuller range of economic measures. Initially, we thought that this finding could be explained by our analytic sampling frame, as partnered men and women may be better able than single people to weather economic stressors because they have a partner to rely on. However, we obtained the same results when we included individuals who were not in a relationship. Another potential explanation for this finding is that there were other factors that offset these economic factors; i.e., income losses due to changes in employment may have been offset by increases in unemployment assistance, policy changes such as the moratorium on evictions or the suspension of student loan payments, or cost savings stemming from lower child care costs or less commuting. Similarly, given the paucity of parental leave in United States, some individuals may have found that job furloughs or greater flexibility in their working conditions provided them with an opportunity to have a child that was otherwise unavailable. Our results are consistent with those of Luppi et al. (2020), who found that the share of respondents in six countries who maintained their fertility plans during the pandemic was not sensitive to their views of the economic implications of the pandemic. Future work should delve more deeply into the economic and employment changes – both good and bad – that have affected the work-family nexus. Further analysis suggested that economic factors were linked to measures of the respondents’ cognitive schema (uncertainty about their relationship, fear of COVID-19 and stress about the future), but were not directly linked to their fertility motivations. Investigating whether economic factors have indirect effects on fertility motivations is an important avenue for future work.

Similarly, the analysis of within-person changes in the desire to avoid a pregnancy showed that these changes were associated with increases in the number of children, lower levels of mental health and higher levels of relationship uncertainty. As in our other analyses, increases in economic stress or in economic hardship were not found to be associated with changes in fertility motivations. These findings highlight that people’s relationships and psychological well-being influence their fertility intentions more than economic factors do. To the extent that the pandemic has led to relationships becoming more uncertain and to increases in depressive symptoms, it is likely that the pandemic will have a negative effect on fertility.

Furthermore, we found evidence that parents were more likely than childless individuals to report an elevated desire to avoid a pregnancy. Given the relatively young age of the analytical sample (in their early to mid-thirties), it may be assumed most of the parents in the sample had school-aged children. This finding likely taps into the stressors that parents faced during the pandemic, as child care centers and schools shut down. For instance, Calarco and colleagues (2020) reported that the increased parenting demands in response to virtual schooling have negatively impacted mothers’ well-being. Although we lacked a sufficient sample size to do so, future work should consider how fertility decision-making during times of uncertainty varies depending on parenthood status, parity and children’s ages.

While this paper has provided new insights into changes in fertility motivations and the underlying factors associated with declines in fertility during the pandemic, it also has a number of limitations. First, most of the respondents in the sample grew up in northwestern Ohio, and their circumstances may not reflect those of the national population. Even though the sample’s demographic characteristics mirror those of a similar cohort at the national level, further analysis of national-level data is warranted. Second, the data were collected before both the major spikes in pandemic-related deaths and the widespread release of vaccines in the U.S. During this period, there were widespread concerns about how best to manage the health and social threats posed by the pandemic. Third, we were unable to determine to what extent fertility would have declined for Americans in this age group in the absence of the pandemic. The decreases we observed may simply reflect the declines that would have otherwise occurred for people in these age groups; however, we lacked the within-person data that we would need to determine whether this was the case. Nonetheless, we were able to account for pandemic-specific factors, and the associations we found between them indicated that the pandemic played some role in these declines. Fourth, the data do not reflect the experiences of a broad age range of adults, as they cover only individuals in their early to mid-thirties. It is possible that younger respondents would have been more responsive to pandemic economic stressors, as they had more time to achieve their fertility goals. Future work should consider more carefully how the pandemic has been experienced by people at different stages of the life course.

While much has been made of changes in the economic realm during the pandemic, it appears that the more proximal influences of the pandemic on fertility motivations were driven by cognitive factors that were linked to worries about falling prey to the coronavirus, relationship strains and stress about the future. These results are in line with the Narrative Framework (Vignoli et al., 2020b,c), which directly assesses how economic constraints frame fertility intentions in Europe. While our results and those of Luppi et al. (2020) hint that economic factors may not be direct drivers of fertility motivations, other studies focusing on the pandemic should further investigate this issue. We argue that our field’s traditional theoretical frameworks may not apply in the same way during the pandemic as they have during other crises, such as the Great Recession. Future work should delve further into the underlying reasons for the changes in fertility motivations by moving beyond established approaches and disciplinary boundaries.

## Figures and Tables

**Table 1:** Distribution of dependent and independent indicators

**Avoiding pregnancy (1–5)**	3.14 (1.74)
Not at all important	30.49%
Not too important	13.24%
Somewhat important	7.32%
Pretty important	9.41%
Very important	39.55%
**Sociodemographic**	
Parent	
No	28.57%
Yes	71.43%
Gender	
Male	40.07%
Female	59.93%
Race/ethnicity	
NH White	72.82%
NH Black	13.94%
Hispanic	11.32%
Other	1.92%
Education	
HS or less	15.85%
Some college	39.72%
College degree	44.43%
Union status	
Dating	12.20%
Cohabiting	25.09%
Married	62.72%
Age (31–38)	34.11 (1.70)
**Pandemic indicators**	
Conservative beliefs	2.97 (1.05)
Fear COVID (1–5)	3.10 (1.01)
Parental disagreements	1.54 (0.77)
Relationship uncertainty (1–5)	1.75 (1.00)
Loss of income	
No	67.49%
Yes	32.51%
Work from home	
No	45.60%
Yes	54.40%
Stress future (1–5)	2.14 (1.02)
Month	
June	37.80%
July	26.48%
August	17.07%
September	12.37%
October/November	6.30%

**Source:** Toledo Adolescent Relationships Study (*n* = 574).

**Table 2:** OLS regression: Importance of avoiding a pregnancy during the pandemic

**Sociodemographic**	
Parent	
(No)	
Yes	0.40*
Gender	
(Male)	
Female	0.11
Race/ethnicity	
(NH White)	
NH Black	−0.09
Hispanic	−0.50*
Other	−1.36*
Education	
(HS or less)	
Some college	0.02
College degree	−0.03
Union status	
(Dating)	
Cohabiting	−0.09
Married	−0.33
Age (31–38)	−0.02
**Pandemic indicators**	
Conservative beliefs (1–5)	−0.02
Fear COVID (1–5)	0.17*
Parental disagreements (1–5)	0.004
Relationship uncertainty (1–5)	0.22**
Loss of income	
(No)	
Yes	−0.24
Work from home	
(No)	
Yes	0.18
Stress future (1–5)	0.17*

**Source:** Toledo Adolescent Relationships Study (*n* = 574).

* *p* < .05

** *p* < .01.

**Table 3:** Distribution of indicators in fixed-effects

	Mean (SD)

**Dependent variable change**	
Change in fertility expectation (−4–4)	0.06 (1.79)
**Independent variable change measures**	
Number of children (0–4)	0.25 (0.59)
Economic hardship (−5–5)	−0.28 (1.26)
Economic stress (−4–3)	−0.16 (1.11)
Physical health (−2–3)	0.03 (0.77)
Mental health symptoms (−56–47)	3.47 (10.54)
Parental closeness (−4–4)	0.01 (0.80)
Relationship uncertainty	−0.63 (2.15)

**Source:** Toledo Adolescent Relationships Study (*n* = 574).

**Table 4:** Fixed-effects of changes in importance to avoid pregnancy

**Change**	
Number of children	0.65**
Economic hardship	0.06
Economic stress	−0.07
Physical health	−0.07
Mental health symptoms	0.02**
Parental closeness	0.02
Relationship uncertainty	0.14**

**Source:** Toledo Adolescent Relationships Study (*n* = 574).

* *p* < .05

** *p* < .01.

## Footnotes

1 At the time of the survey, the TARS cohort studied here was aged 29–36, and many its members were already parents. Thus, it is likely that they believed they had sufficient time to reach overall fertility goals, if they had not met them already.
